# A systematic review of the use of financial incentives and penalties to encourage uptake of healthy behaviors: protocol

**DOI:** 10.1186/2046-4053-1-51

**Published:** 2012-10-31

**Authors:** Jean Adams, Emma L Giles, Shannon Robalino, Elaine McColl, Falko F Sniehotta

**Affiliations:** 1 Institute of Health & Society, Baddiley-Clark Building, Richardson Road, Newcastle upon Tyne, NE2 4AX, UK; 2 Institute of Health & Society, Newcastle Clinical Trials Unit, 4th Floor, William Leech Building, The Medical School, Framlington Place, Newcastle upon Tyne, NE2 4AH, UK

**Keywords:** Behavior change, Financial incentives, Systematic review, Health behaviors, Public health

## Abstract

**Background:**

The use of financial incentives and penalties to encourage uptake of healthy behaviors is increasingly seen as a viable intervention in developed countries. Previous reviews of the effectiveness of financial incentives and penalties for encouraging the uptake of healthy behaviors have focused on individual behaviors making it difficult to draw overall conclusions about the effectiveness of such interventions. This systematic review will explore the effectiveness of financial incentives and penalties for encouraging a wide range of behaviors, including: smoking cessation, increased physical activity, healthier dietary intake, sensible patterns of alcohol consumption, safe sun, safe sex, and primary preventive clinical behaviors.

**Methods:**

Systematic methods will be used to search existing literature and screen studies for inclusion. All studies that meet the following inclusion criteria will be included in the review: participants were 18 years old or older and living in high-income countries; interventions included cash or cash-like incentives to promote the uptake of healthy behaviors, or cash or cash-like penalties to discourage unhealthy behaviors; the comparator was usual care or no intervention; study design was randomized controlled trial, cluster randomized controlled trial, controlled before and after study, or interrupted time series analysis. Two reviewers will independently screen the publications to ensure they meet the inclusion criteria. Quality will be assessed by two researchers, working independently, using the Cochrane risk of bias tool. Meta-analysis will be conducted, if appropriate. Any studies identified as at ‘high risk of bias’ will be excluded from meta-analysis.

**Discussion:**

This systematic review will provide policy-relevant recommendations for the use of financial incentives and penalties as a method of encouraging uptake of healthy behaviors.

## Background

Health-promoting behaviors include the ‘big four’ behaviors of not smoking, sensible alcohol consumption, eating a ‘healthy’ diet, and taking regular physical activity; as well as safe sun and sex behaviors and primary preventive clinical behaviors, such as attending for vaccinations and screening. Poor engagement in these behaviors is a key determinant of morbidity and mortality and results in substantial social, healthcare and economic costs. Despite consistent efforts to encourage uptake of healthy behaviors [1,2], unhealthy behaviors remain common.

In general, behavioral incentives have been defined as motivating rewards that are provided contingent on behavioral performance [3]. However, this definition could be interpreted as including any reward (for example, a sticker or praise) and not just financial rewards. Furthermore, this definition specifically excludes the converse of motivating rewards - penalties. While non-financial rewards may also increase health promoting behaviors, it is highly likely that in advanced societies, interventions offering financial rewards are conceptually different from those offering rewards with social, emotional or tokenistic value. As such, this review is restricted to financial incentives and penalties and we define these as interventions that offer cash or cash-like rewards (for example, vouchers that can be exchanged for goods or services) contingent on performance of the target healthy behavior, but widen this to include interventions imposing cash or cash-like (for example, reductions in welfare benefits) penalties contingent on non-performance of the target healthy behavior. Hence forth we use the term ‘financial incentives’ to refer to both these incentives and penalties.

The reasons why individuals pursue unhealthy behaviors are diverse and include environmental, social, psychological and socio-demographic factors [4]. One further reason relates to the relative balance and timing of the costs and rewards of engaging in these behaviors, and personal preferences for these (that is, ‘time preference’). Behavioral economic theory suggests that individuals commonly hold inconsistent preferences for similar outcomes occurring at different points in the future, with outcomes in the near future generally valued more than those in the distant future [5]. While the health gains of health-promoting behaviors are often delayed in time, the financial and opportunity costs can be immediate. As these immediate costs are ‘dis-valued’ more than the delayed health benefits are valued, individuals make a ‘rational’ choice to pursue unhealthy behaviors. Providing immediate rewards for performance of health behaviors, or penalties for non-performance is likely to change the temporal reward structure associated with these behaviors, working with, rather than against, individuals’ time preferences [6,7].

Using financial incentives to encourage uptake of healthy behaviors may appear to be a simple solution to a serious public health problem. However, these interventions are not simple. They differ in terms of the behavior incentivized; the value, nature (that is, reward or penalty; cash or voucher) and timing (that is, immediately following, or remote from, performance of the behavior) of the incentive; as well as the other behavioral change techniques that may be incorporated into the programs of which they are a part (for example, keeping a record of the target behavior or agreeing to a behavioral contract) [8]. While there is a growing range of evaluations of financial incentives for encouraging uptake of healthy behaviors [9-14], conclusions on what makes an effective financial incentive program in this context remain limited to the suggestion that incentives are more useful for encouraging simple one-off behaviors, such as attendance for vaccinations, than more complex sustained behavior changes, such as smoking cessation [7,13,15]. In addition, variations in the effectiveness of financial incentives across population groups have not been explored. The apparent failure of many financial incentive programs to achieve sustained behavioral change may reflect sub-optimal design of the intervention, rather than a failure of incentives *per se*.

A number of systematic and non-systematic reviews have been conducted in this area [9-14,16,17]. However, most focus on individual behaviors (for example, smoking or weight loss), rather than exploring the full range of healthy behaviors, or are restricted to developing countries where absolute financial hardship may be much more common [16]. The current review will fill this gap by conducting a systematic review of primary studies which explore the use of financial incentives to encourage uptake of the full range of healthy behaviors. As described below, systematic procedures will be used to search the literature and identify reports of research for inclusion. An established tool for assessing the quality of included reports will be used to grade the quality of existing evidence. A narrative and tabular summary of the existing evidence will be produced. The potential for meta-analysis will be explored and this will be conducted if appropriate.

The UK Secretary of State for Health has signaled his interest in using financial incentives for encouraging uptake of healthy behaviors [18] and the UK National Institute for Health and Clinical Excellence is consulting on the topic [19]. This research will provide policy-relevant information for the design of evidence-based financial incentive interventions which can be subjected to robust evaluation.

### Research questions

This systematic review will answer the following primary research question:

What is the effectiveness of financial incentives for encouraging uptake of healthy behaviors among adults living in high-income countries?

Secondary research questions are:

1. What formats do financial incentives in included studies take (for example, cash, coupons, vouchers, pay deductions)?

2. What is the range of healthy behaviors that have been targeted (for example, smoking, physical activity, dietary behaviors, alcohol consumption, safe sun, safe sex, and primary preventive clinical behaviors)?

3. Apart from the incentive component, what other behavior change techniques are used as part of these interventions?

4. What theoretical rationales have been used to guide intervention development?

5. Does effectiveness of financial incentive programs vary according to:

 a. the length of the intervention period?

 b. the length of the follow up period, after the active intervention ceases?

 c. the format of the incentive (for example, cash, voucher, reward, penalty, total value)?

 d. the nature of the behavior incentivized (for example, one-off, sustained behavior change)?

 e. socio-demographic characteristics (for example, age, gender, socio-economic position) of recipients?

 f. inclusion of other behavior change techniques in the intervention program?

## Methods/design

### Overview of the search strategy

The search strategy will focus on research reports, primarily identified through automated searching of publications databases. Trial registers will also be searched and the reference lists of previous related systematic reviews [9-14,16] scanned for relevant articles. Attempts will be made to access unpublished reports and those published in the grey literature by using internet search engines and sending requests to appropriate email discussion lists and organizations. There will be no systematic hand searching of journals or conference proceedings.

### Inclusion criteria

All identified studies that meet the following criteria will be included in the review:

• Language: Have an English language title and abstract.

• Date range: All dates.

• Study design: Randomized controlled trials, cluster randomized controlled trials, controlled before and after studies or interrupted time series analyses.

• Population: Adults (18 years old or older) living in high-income economies (those with a Gross National Income of $12,276 or more per capita in 2010, as identified by the World Bank).

• Intervention: Financial incentives designed to encourage uptake of health behaviors at the individual level; defined as: cash or cash-like rewards (or penalties) that are provided contingent on change (or non-change) in a specific healthy behavior (for example, increase in physical activity).

• Target behavior: Smoking cessation; increase in physical activity; adoption of sensible patterns of alcohol consumption; increase in consumption of ‘healthy’ foods such as fruit and vegetables; decrease in consumption of ‘unhealthy’ foods such as those high in fat, salt or sugar; and attendance for screening or vaccination

• Comparator: Usual care, or no intervention.

• Outcome measure: Objective or self-report measures of healthy behaviors that have been validated against objective measures.

We have restricted the search to studies with an English language title and abstract as we do not have resources for substantial translation costs. However, the majority of the databases we will search contain only English language material and in most cases where English language full-text is not available, titles and abstracts will be provided in English.

The review is restricted to adult populations as the use of financial incentives to encourage uptake of healthy behaviors in young people has been systematically reviewed elsewhere [20]. Interventions aimed at parents and using financial incentives to encourage them to take action to improve their children’s health behaviors do not specifically require health behavior change in the individuals receiving the incentive and do not, therefore, meet the inclusion criteria for the review.

### Search strategies

The following sources will be searched to identify studies that meet the inclusion criteria:

• Electronic databases of peer-reviewed journal articles; including those covering biomedicine (that is, Medline, Embase, Science Citation Index), nursing and allied health professions (that is, CINAHL) and the social sciences (that is, Social Science Citation Index, PsycInfo, Applied Social Science Index and Abstracts, and the International Bibliography for the Social Sciences).

• Online research registers; including trial registers (that is, Current Controlled Trials, clinicaltrials.gov) and systematic review registers (that is, Cochrane Library of Systematic Reviews, Database of Abstracts of Reviews of Effects).

• The reference lists of all studies that meet the inclusion criteria, as well as relevant reviews, will be scanned to identify further relevant publications.

• Citation searches of all studies that meet the inclusion criteria will be performed in the Science and Social Science Citation Indices to identify other relevant publications.

More details of the search strategy are provided in the Additional file 1.

### Ancillary search procedures

As the inclusion criteria are restricted to randomized controlled trials, cluster randomized controlled trials, interrupted time series analyses, and controlled before and after studies, it is not anticipated that there will be a large body of grey literature. In addition to searching trial registers, unpublished, or grey, literature will be accessed by sending emails to relevant on-line discussion lists and organizations covering topics including public health and preventive medicine (for example, public-health@jiscmail.ac.uk; US Preventive Services Task Force), incentives for health (for example, health-incentives@jiscmail.ac.uk), and evidence synthesis (for example, Cochrane Effective Practice and Organisation of Care Group, Cochrane Public Health Group, The Joanna Briggs Institute) requesting that any unpublished reports are forwarded to the study team.

### Screening

After importing search results into EndNote and removing duplicates, screening will be conducted in two phases. Firstly, titles and abstracts will be screened by one researcher (ELG) to identify publications that definitely do not meet the inclusion criteria. In any cases of doubt, publications will be included.

Secondly, the full text of the publications that were included following the first screening will be screened by two researchers (ELG and JA), working independently. On this occasion the assessment will be whether publications meet the inclusion criteria. Any disagreements at this stage will be resolved by discussion between researchers and then with the full study team. Tables of excluded studies will be prepared, detailing when exclusion occurred and the reasons for exclusion.

### Data extraction

A coding framework will be developed in an appropriate software package. This will be used to record details of, as appropriate: participant characteristics, setting, time period, intervention, comparator, outcome, study design, method of analysis, factors considered in analysis, results, and quality assessment. Data will be extracted by one reviewer and checked by a second. Any disagreements will be resolved by discussion. The behavior change techniques used alongside the financial incentives will be assessed using Michie *et al.*’s (2011) revised taxonomy of behavioral change techniques [21].

As we will make efforts to access grey literature, it is likely that there will be cases where we retrieve both an internal report and a peer-reviewed paper on the same study. In these cases, peer-reviewed findings will be favored, although additional details provided in the internal report will be extracted if relevant. Where publications lack details required for quality assessment or full data extraction, authors will be contacted to request further details.

### Risk of bias/quality assessment

The quality of all studies that meet the inclusion criteria will be formally assessed by two researchers working independently using the extension of the Cochrane risk of bias tool [22] for randomized controlled trials, non-randomized controlled trials and interrupted time series analyses suggested by the Cochrane Effective Practice and Organisation of Care Group [23]. This information will be used to describe the quality of available research and to conduct sensitivity analysis in any meta-analyses. Any studies with a ‘high risk of bias’ will be excluded from any meta-analyses during sensitivity analysis.

### Strategy for data synthesis and reporting

We will begin by describing the range of interventions (both financial incentive and other behavior change technique components), study designs, theoretical rationales used to guide incentive development, population characteristics, and the behavioral outcomes that have been studied. The length of the intervention and follow-up periods will be presented overall, as well as the nature of the incentive, the nature of the behavior incentivized and the size of the reported effects. These will be grouped according to population, intervention type and outcome, with the results summarized in tabular form.

The potential for meta-analyses, using random or fixed effects models as appropriate, will be explored and conducted if applicable. If appropriate, separate meta-analyses will be conducted for different types of study designs, interventions and outcomes. Funnel plots will be used to explore publication bias. Sensitivity analysis will explore the effect of excluding studies that appear to be statistical outliers, and those of poorer methodological quality. All analyses will be conducted in RevMan 5.1. As previously described [24], the number and type of behavioral change techniques used in each study will be documented and compared to effect size to determine if any clear patterns emerge.

Finally, we will prepare a ‘summary of findings’ table as described in the Cochrane Handbook [22] and use the Grading of Recommendations Assessment, Development and Evaluation Working Group approach for describing the quality of relevant evidence [25].

## Discussion

This systematic review will provide policy-relevant recommendations for the use of financial incentives as a method of encouraging uptake of healthy behaviors.

## Abbreviations

RevMan: Review Manager.

## Competing interests

The authors declare that they have no competing interests.

## Authors’ contributions

JA conceived the idea for the study, obtained funding, drafted the protocol and will act as second reviewer. ELG revised the protocol, will act as first reviewer and prepare results for reporting. SR devised the search strategy. EM and FS contributed to protocol development and will contribute to data interpretation. All authors read and approved the final manuscript.

## Supplementary Material

Additional file 1 Search strategy for identification of studies for financial incentives. Click here for file
