# Supplementary material for: A systematic review of the use of financial incentives and penalties to encourage uptake of healthy behaviors: protocol
Source: Syst Rev. 2012 Oct 31;1:51. doi: 10.1186/2046-4053-1-51 (PMC3499145; doi:10.1186/2046-4053-1-51)
Supplement: Additional file 1 — Search strategy for identification of studies for financial incentives. [file 2046-4053-1-51-S1.docx]

**Search strategy for identification of studies for financial incentives**

**Databases**

The following electronic databases were searched for relevant papers:

- ASSIA (1987-April 2012)
- CINAHL (1981-April 2012)
- EMBASE (1980-April 2012)
- IBSS (1951-April 2012)
- MEDLINE (1946-April 2012)
- PsycINFO (1806-April 2012)
- Science Citation Index (1970-April 2012)
- Social Science Citation Index (1970-April 2012)
- The Cochrane Library (DARE, CDSR, CENTRAL, NHS EED, HTA) (?-April 2012)

**Search terms**

Search terms were agreed following a scoping search undertaken by the information specialist and discussions with the review team. The search was split into three concepts: incentives, behaviour change, and behaviours. The search was limited to papers published in English, but no further limits (e.g. date or study design) were placed on the search. The following sets list the terms used across the databases. The terms were combined together along with appropriate thesaurus terms and truncation appropriate to individual databases.

Set 1: incentives

Conditional cash, contingency management, financial incentive, gift, gift certificate, money, paying patient, tax credit, award, benefit, cash, competition, contest, coupon, discount, disincentive, forfeit, incentive, inducement, lottery, monetary, nonmonetary, payment, penalisation/penalization, penalise/penalize, penalty, prize, reinforcement, relinquish, reward, taxation, taxes, token, voucher, pay deduction, P4P4P, Pay-For-Performance For Patients

Set 2: behaviour change

Attitude-change, attitude, behaviour, behaviour near change, behaviour near intervention, behaviour near modification, behaviour near risk, behaviour near therapy, health near behaviour, health near attitude, life style, life-style, lifestyle, abstinence, prevention, preventative

Set 3: behaviours

5 a day, five a day, alcohol, cigarette, diet, drinking, exercise, healthy diet, healthy food, healthy habit, healthy eating, immunisation/immunization, immunise/immunize, nutrition, physical activity, screening, smoking, stopping smoking, smoking cessation, tobacco, vaccinate, vaccination, weight loss, weight maintenance, weight management, weight reduction

(A sample search is included in the appendix. Search strategies for individual databases are available upon request.)

**Example search**

**MEDLINE**

This search was translated as appropriate in other databases.

1. Gift Giving/

2. "Cost Savings"/

3. *motivation/

4. exp reward/

5. *punishment/

6. 1 or 2 or 3 or 4 or 5

7. (conditional adj cash).ab,ti.

8. (contingency adj management).ab,ti.

9. (financial adj incentive$).ab,ti.

10. gift.ab,ti.

11. (gift adj certificate).ab,ti.

12. money.ab,ti.

13. "pay$ adj2 patient".ab,ti.

14. (tax adj credit).ab,ti.

15. "benefit$".ab,ti.

16. cash.ab,ti.

17. "competition$".ab,ti.

18. "contest$".ab,ti.

19. coupon.ab,ti.

20. discount.ab,ti.

21. "disincentiv$".ab,ti.

22. "forfeit$".ab,ti.

23. "incentiv$".ab,ti.

24. "inducement$".ab,ti.

25. "lotter$".ab,ti.

26. monetary.ab,ti.

27. (non-monetary or nonmonetary).ab,ti.

28. payment.ab,ti.

29. (penalization or penalisation).ab,ti.

30. (penalize or penalise).ab,ti.

31. "penalt$".ab,ti.

32. "prize$".ab,ti.

33. reinforcement.ab,ti.

34. "relinquish$".ab,ti.

35. "reward$".ab,ti.

36. taxation.ab,ti.

37. taxes.ab,ti.

38. "token$".ab,ti.

39. "voucher$".ab,ti.

40. ((P4P4P or Pay-For-Performance) adj2 Patients).ab,ti.

41. (pay adj deduction$).ab,ti.

42. 7 or 8 or 9 or 10 or 11 or 12 or 13 or 14 or 15 or 16 or 17 or 18 or 19 or 20 or 21 or 22 or 23 or 24 or 25 or 26 or 27 or 28 or 29 or 30 or 31 or 32 or 33 or 34 or 35 or 36 or 37 or 38 or 39 or 40 or 41

43. 6 and 42

44. exp attitude to health/

45. *harm reduction/

46. health behavior/

47. exp health promotion/

48. exp life style/

49. *occupational health/

50. *patient compliance/

51. exp preventive health services/

52. *risk reduction behavior/

53. occupational health services/

54. 44 or 45 or 46 or 47 or 48 or 49 or 50 or 51 or 52 or 53

55. "attitude$".tw.

56. "behavio$".tw.

57. (health adj attitude$).tw.

58. ((life adj style) or life-style or lifestyle).tw.

59. abstinence.tw.

60. "preventi$".tw.

61. preventative.tw.

62. 55 or 56 or 57 or 58 or 59 or 60 or 61

63. 54 or 62

64. 43 and 63

65. smoking/pc

66. exp tobacco/

67. exp "Tobacco Use Cessation"/

68. *drinking behavior/

69. *alcohol drinking/

70. substance-related disorders/

71. *food habits/

72. diet/

73. weight loss/

74. exp immunization/

75. *mass screening/

76. exp exercise/

77. 65 or 66 or 67 or 68 or 69 or 70 or 71 or 72 or 73 or 74 or 75 or 76

78. alcohol.tw.

79. "cigarette$".tw.

80. (diet adj behavio$).tw.

81. drinking.tw.

82. exercise.tw.

83. (healthy adj diet).tw.

84. (healthy adj food).tw.

85. (healthy adj habit).tw.

86. (healthy adj eating).tw.

87. (immunisation or immunization).tw.

88. (immunise or immunize).tw.

89. "nutrition$".tw.

90. (physical adj activity).tw.

91. screening.tw.

92. smoking.tw.

93. tobacco.tw.

94. "vaccinat$".tw.

95. (weight adj loss).tw.

96. (weight adj maintenance).tw.

97. (weight adj management).tw.

98. (weight adj reduction).tw.

99. 78 or 79 or 80 or 81 or 82 or 83 or 84 or 85 or 86 or 87 or 88 or 89 or 90 or 91 or 92 or 93 or 94 or 95 or 96 or 97 or 98

100. 77 or 99

101. 64 and 100

102. limit 101 to humans
